# Cytotoxic Activity of the Histone Deacetylase 3-Selective Inhibitor Pojamide on MDA-MB-231 Triple-Negative Breast Cancer Cells

**DOI:** 10.3390/ijms20040804

**Published:** 2019-02-13

**Authors:** Claudio Luparello, Dalia Maria Lucia Asaro, Ilenia Cruciata, Storm Hassell-Hart, Supojjanee Sansook, John Spencer, Fabio Caradonna

**Affiliations:** 1 Dipartimento di Scienze e Tecnologie Biologiche, Chimiche e Farmaceutiche (STEBICEF), Università di Palermo, Viale delle Scienze, 90128 Palermo, Italy; daliaasaro@libero.it (D.M.L.A.); ilenia.cruciata@unipa.it (I.C.); fabio.caradonna@unipa.it (F.C.); 2 Department of Chemistry, School of Life Sciences, University of Sussex, Falmer, Brighton BN1 9QJ, UK; S.Hassell-Hart@sussex.ac.uk (S.H.-H.); sansook_s@yahoo.com (S.S.); j.spencer@sussex.ac.uk (J.S.)

**Keywords:** histone deacetylase inhibitor, breast cancer cells, cell viability, cell cycle, apoptosis, autophagy, reactive oxygen species, mitochondrial transmembrane potential

## Abstract

We examined the effects of the ferrocene-based histone deacetylase-3 inhibitor Pojamide (*N*^1^-(2-aminophenyl)-*N*^8^-ferrocenyloctanediamide) and its two derivatives *N*^1^-(2-aminophenyl)-*N*^6^-ferrocenyladipamide and *N*^1^-(2-aminophenyl)-*N*^8^-ferroceniumoctanediamide tetrafluoroborate on triple-negative MDA-MB-231 breast cancer cells. Viability/growth assays indicated that only the first two compounds at 70 μM concentration caused an approximate halving of cell number after 24 h of exposure, whereas the tetrafluoroborate derivative exerted no effect on cell survival nor proliferation. Flow cytometric and protein blot analyses were performed on cells exposed to both Pojamide and the ferrocenyladipamide derivative to evaluate cell cycle distribution, apoptosis/autophagy modulation, and mitochondrial metabolic state in order to assess the cellular basis of the cytotoxic effect. The data obtained show that the cytotoxic effect of the two deacetylase inhibitors may be ascribed to the onset of non-apoptotic cell death conceivably linked to a down-regulation of autophagic processes and an impairment of mitochondrial function with an increase in intracellular reactive oxygen species. Our work expands the list of autophagy-regulating drugs and also provides a further example of the role played by the inhibition of autophagy in breast cancer cell death. Moreover, the compounds studied may represent attractive and promising targets for subsequent molecular modeling for anti-neoplastic agents in malignant breast cancer.

## 1. Introduction

Histone deacetylases (HDAC) are enzymatic epigenetic modulators whose involvement in the modification of chromatin structure and regulation of the activity of many non-histone proteins is widely recognized. They are grouped into four classes on the basis of functional criteria and homology to yeast proteins; they show distinct gene expression patterns, intracellular localization, and role [1]. HDAC inhibition is known to affect several intracellular processes, such as gene expression, signal transduction, and protein turnover, and to ultimately alter the proliferation, survival, and immunogenicity of cancer cells. Within this context, HDAC inhibitors (HDACi) are an emerging class of heterogeneous compounds that are grouped into different categories on the basis of their chemical nature. Their specific activity may be directed to all or a broad spectrum of HDAC classes, such as the “pan”-inhibitor SAHA, or effects may be isoform specific [2]. Although only four HDACis have been approved by the U.S. Food and Drug Administration, a great number of them are undergoing clinical trials in order to thoroughly exploit their full potential as anticancer agents [3,4]. Increasing knowledge of the distinct pathways that are more specifically involved in tumor growth or metastasis in different neoplastic histotypes has emphasized the need to develop selective agents that target individual HDACs and the investigation, at the molecular level, of the biological response to such selective inhibitors by cancer cells.

Triple-negative breast cancers (TNBC) do not show estrogen and progesterone receptor expression. Also being HER2/neu negative, they are refractive toward hormone-based therapies and to drugs that target HER2. This renders the TNBC cytotype particularly “aggressive” and potentially highly malignant, thereby being associated usually with a worse prognosis than other breast tumor types [5]. The limitation of treatment options against TNBC has prompted the development of novel compounds or analogs of pre-existing compounds that might counteract neoplastic cell growth but that, conversely, require substantial biological characterization. The MDA-MB-231 cell line has been selected as a model system herein. It originates from a pleural effusion of a basal subtype TNBC and exhibits many aspects of a highly malignant phenotype, such as the inactivation of p53 due to a mutation in codon 280 of exon 8 and the ability to form metastases in vivo [6,7,8].

**Figure 1:**
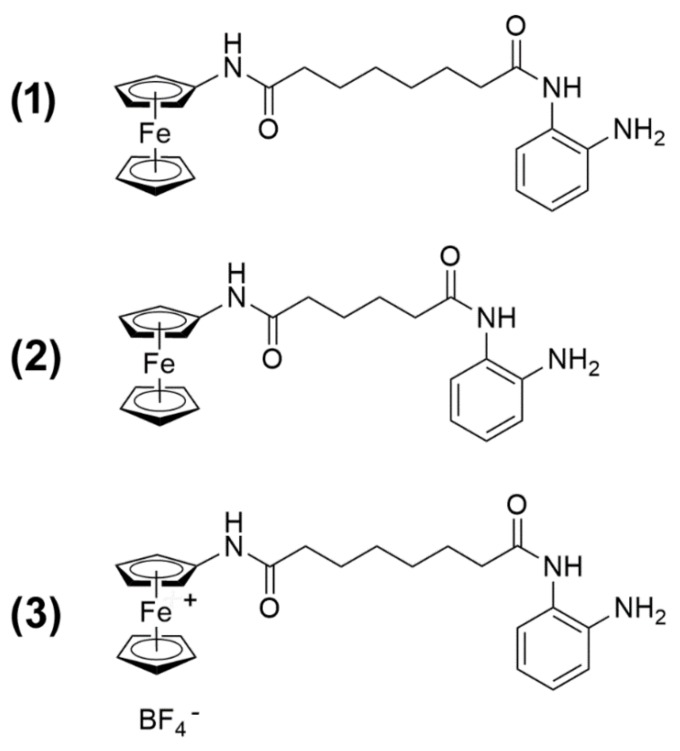
Compounds used in the present study.

In a previous publication, we [9] reported the synthesis of the novel HDACi Pojamide (*N*^1^-(2-aminophenyl)-*N*^8^-ferrocenyloctanediamide, 1), a ferrocene-containing *o*-aminoanilide that can be activated in cells to a Fe^III^ species. This compound displays an optimal arrangement of a ferrocene cap—which can be oxidized—a linker, and a benzamide zinc-binding motif, as opposed to the more common hydroxamic acid, to enable selective inhibitory activity toward HDAC3, one of the major class I HDACs. Overexpression of this isoform has been reported in a broad range of neoplastic histotypes, and its activity was found related to a variety of aspects of tumor progression. In particular, in TNBC, it appeared to be involved in stimulating cell migration and maintaining the cancer stem cell subpopulation via the Akt–GSK3β–β-catenin signaling pathway [10,11]. The goal of the present study was to thoroughly examine the effects of **1** on TNBC cells of the MDA-MB-231 line with respect to viability and proliferation, cell cycle distribution, apoptosis and autophagy modulation, and mitochondrial metabolic/cell redox state. In addition, a comparative evaluation was made with two derivatives of **1**: *N*^1^-(2-aminophenyl)-*N*^6^-ferrocenyladipamide (2) and *N*^1^-(2-aminophenyl)-*N*^8^-ferroceniumoctanediamide tetrafluoroborate (3) (Figure 1). Compound **2** contains a four-carbon aliphatic chain, thereby resulting in a shorter “linker” portion of the molecule with a slightly shifted enzyme docking with respect to 1 and, as a consequence, weaker HDAC3 activity. Compound **3** is a preformed, independently synthesized, oxidized analog of 1. Such a Fe^III^ derivative is postulated to be formed in 1-treated cells when sodium nitroprusside oxidant is added, but it is a charged species and, therefore, is supposedly poorly permeable in cells if administered exogenously [9].

## 2. Results

**Figure 2:**
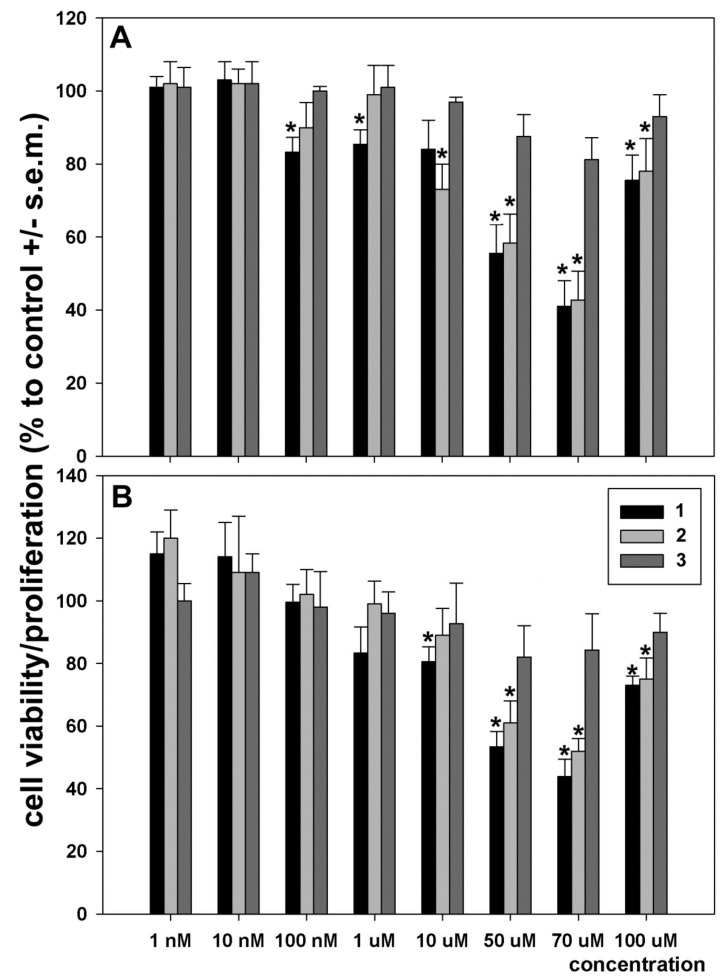
Effect of 24 h incubation with **1**, **2,** and **3** on the viability of MDA-MB-231 cells. (**A**) Dose–response bars from quadruplicate cell counting assays. (**B**) Dose–response bars from quadruplicate MTS assays. Four replicates were run for each assay. The results are expressed as the mean ± standard error of the mean (SEM). * *p* < 0.05.

In the first set of experiments, we checked the effect of dose- and time-dependent incubation with either **1**, **2,** or **3** on MDA-MB-231 cell viability via direct cell counting and MTS assay. As shown in Figure 2, when cells were exposed for 24 h to **1** and **2,** these compounds caused a decrease in cell number starting from 10 nm and 10 μM concentrations for **1** and **2**, respectively. A dose–response U-shaped curve is evident with a maximal effect equal to 70 μM for both compounds, among the concentrations tested. Since exposure to the two molecules for longer times (i.e., 48 and 72 h) did not appear to substantially modify the effect observed after 24 h of incubation, the concentration of 70 μM and the duration of exposure of 24 h were chosen for all the subsequent biochemical experiments. On the other hand, exposure to **3** at any concentration tested did not exert significant effects on MDA-MB-231 cell viability and, therefore, this compound was not submitted to further analyses.

For comparative and more detailed insight into the cytotoxic action of **1** and **2** on triple-negative MDA-MB-231 cells, cell cycle state, apoptosis induction markers (phosphatidylserine externalization and caspase 8 activation), autophagy markers (quantitation of acidic vesicular organelles (AVOs), and intracellular accumulation of p62/Sequestosome-1 (SQSTM1), Beclin-1 and microtubule-associated protein light chain-3 (LC3)-I and -II), and markers of mitochondrial metabolism/cell redox state (mitochondrial transmembrane potential (MMP) and reactive oxygen species (ROS) production) were studied.

**Figure 3:**
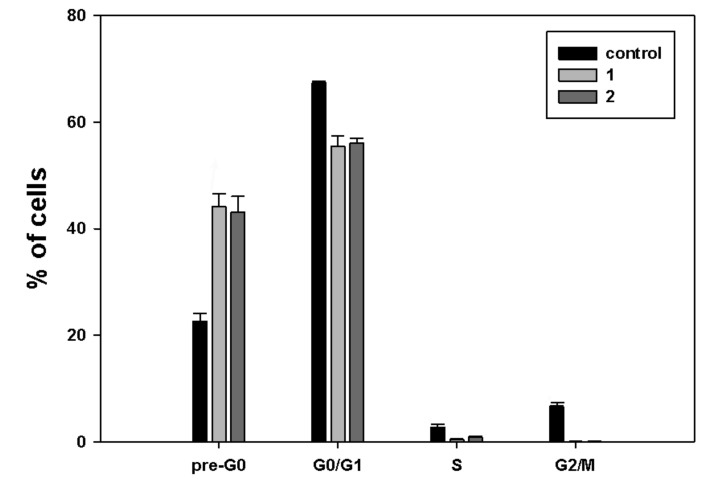
Cell cycle distribution of MDA-MB-231 cells exposed to **1** and **2**, compared to control conditions. The results are expressed as the mean ± SEM. of triplicate assays. Four replicates were run for each assay. All *p* values were <0.05 if compared to controls.

First, in order to study the effects induced by treatment with **1** and **2** on MDA-MB-231 cell cycle, cells treated with the HDACi for 24 h were stained with propidium iodide and studied by flow cytometric analysis for the distribution of cell cycle phases. Figure 3 shows that exposure to both **1** and **2** was chiefly linked with a higher percentage of cells in the pre-G_0_ fraction (44.1% and 43.1%, respectively, vs. 22.6% of control), consistent with an increase in damaged and fragmented cells due to cytotoxicity of the compounds.

A more or less pronounced decrease in the cell fractions was found in the other cell cycle phases: in particular, the G_0_/G_1_ phase fraction of **1** and **2** accounted for 55.4% and 56%, respectively, vs. 67.3% of control, the S phase fraction for 0.4% and 0.8%, respectively, vs. 3% of control, and the G_2_/M phase fraction for 0.1% for both molecules vs. 6.6% of control. 

**Figure 4:**
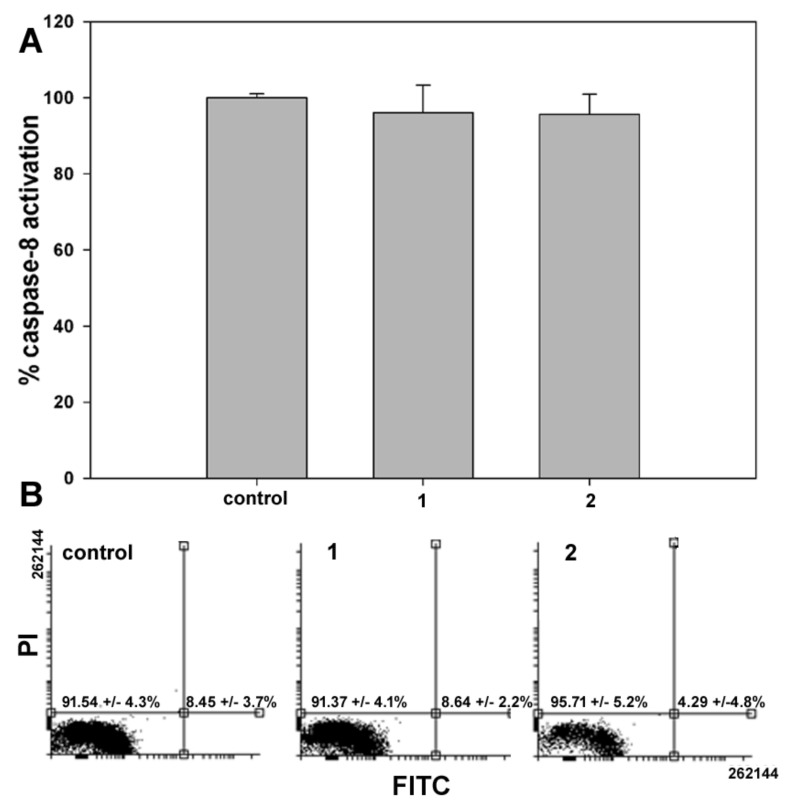
Representative flow cytometric assays for apoptosis in MDA-MB-231 cells cultured in control conditions or exposed to either **1** or **2** for 24 h. Evaluations of the extent of (**A**) caspase-8 activation using the Vybrant® FAM Caspase-8 Assay Kit, and (**B**) phosphatidylserine externalization using the Annexin V apoptosis Detection Kit. In (**B**), the dot plots show the result of a representative experiment and the percentages indicated in the left and right quadrants refer to live annexin V-/propidium iodide cells and early apoptotic annexin V+/propidium iodide cells, respectively. The results are expressed as the mean ± SEM of triplicate assays.

In the second set of experiments, the onset of apoptosis, if any, in samples of control and **1**- or **2**-treated cells was checked through two different assays having phosphatidylserine externalization and caspase-8 activation as endpoints. As shown in Figure 4, data from both assays showed no difference between control and exposed cells, confirming that programmed cell death was not involved in **1** and **2** cytotoxicity after 24 h treatment. Comparable results were obtained with shorter exposure to the compounds (6 and 12 h).

**Figure 5:**
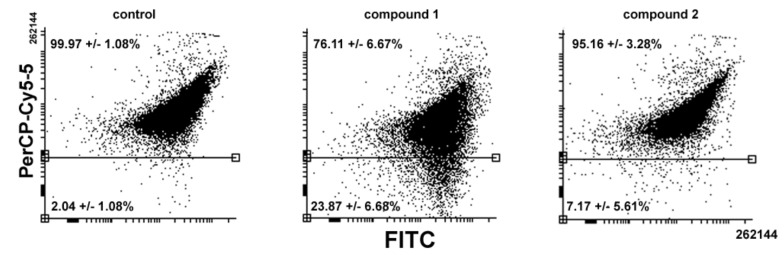
Representative flow cytometric analyses of AVO accumulation in MDA-MB-231 cells cultured in control conditions or exposed to either **1** or **2** for 24 h. The percentage indicated in the top quadrants refers to AVO-positive cells. The dot plots show the result of a representative experiment. The results are expressed as the mean ± SEM of triplicate assays.

It is known that the autophagy rate of MDA-MB-231 cells is constitutively high [12], hereby furnishing cells with energy and the basic elements in order to counterbalance the metabolic stress due to oxygen and nutrient shortage during fast proliferation. Moreover, it is recognized that autophagy down-regulation sensitizes MDA-MB-231 tumor cells to the cytotoxic effect of chemical and physical agents [13,14]. Therefore, we firstly checked via acridine orange staining whether **1** and **2** might lead to a modification in autolysosome numbers, also known as AVOs, a hallmark of autophagy. Figure 5 shows that **1**-treated cells led consistently to a reduction in the amount and size of AVOs whereas exposure to **2** showed a more limited decrease compared to the control (**1** vs. **2** vs. control = 76.11% vs. 95.16% vs. 99.97%).

**Figure 6:**
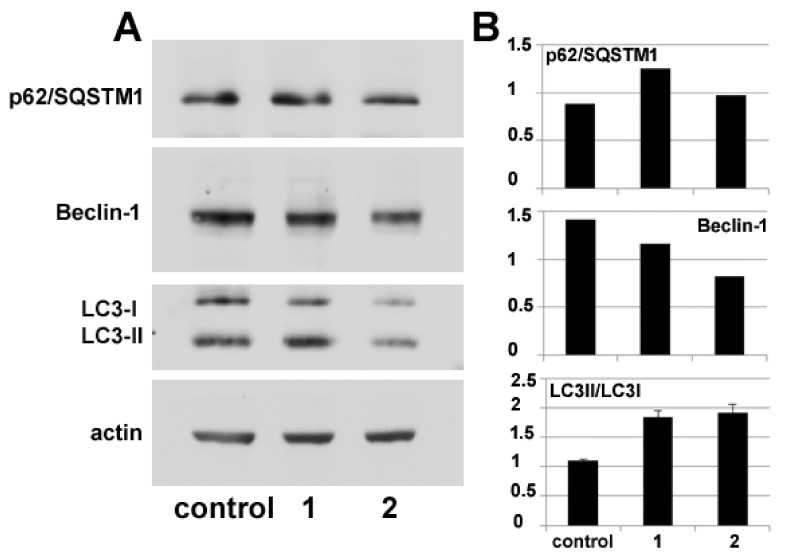
(**A**) Representative immunoblot analysis of autophagy molecular markers in MDA-MB-231 cells cultured in control conditions or exposed to either **1** or **2** for 24 h. (**B**) Histograms showing the relative extent of marker quantitation after normalization with actin as the loading control. The results are expressed as the mean ± SEM of triplicate assays. All *p* values were <0.05 if compared to controls.

Autophagy modulation by **1** and **2** treatment was also verified via molecular markers through protein blot analysis of the intracellular accumulation levels of Beclin-1 and p62/ SQSTM1, whose variations are used to monitor the onset of autophagy, and of the conversion of LC3 from its cytosolic form (LC3-I) to its autophagosome-associated lipidated form (LC3-II), which can be identified electrophoretically due to its lower molecular mass [15]. The immunoblots and the histograms in Figure 6 show that the accumulation of autophagy markers is modified by exposure to the compounds. In particular, p62/SQSTM1 amount appears to increase after 24 h of incubation with **1** and **2** although in the latter case the augmentation is fainter. In parallel, the amount of Beclin-1 decreases, more prominently in the presence of **2**, and LC3-II/LC-3I ratio increases in HDACi-treated cells with respect to the control.

**Figure 7:**
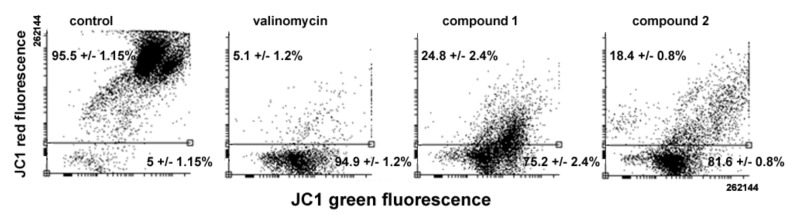
Representative flow cytometric analysis of MMP in MDA-MB-231 cells cultured in control conditions, or exposed to either valinomycin (as a positive control), **1,** or **2** for 24 h. The percentages indicated in the bottom quadrants in each frame quantitate the amount of low red-emitting cells that underwent dissipation of MMP. The results are expressed as the mean ± SEM of triplicate assays.

**Figure 8:**
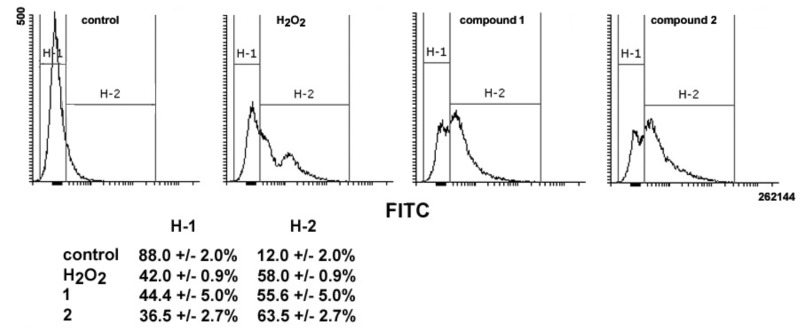
Representative histograms showing ROS generation in MDA-MB-231 cells cultured in control conditions or exposed to either H_2_O_2_ (as a positive control), **1**, or **2** for 24 h. The x-axis reports the intensity of green fluorescence emitted by the dye 2’,7’-dichlorodihydrofluorescein, which is proportional to the amount of cytosolic ROS. The fluorescence percentages of the gated regions H-1 and H-2 of the histograms are reported under the graphs. The results are expressed as the mean ± SEM of triplicate assays.

Variations in MMP levels following cell exposure to the drugs were verified using the JC1 probe. As shown in Figure 7, flow cytometry analysis evidences a loss of MMP in treated cells, the percentage of low red-emitting cells (bottom quadrant) being about 75% and 81% after 24 h of exposure to **1** and **2**, respectively, vs. approximately 5% of control cells. It is known that the oxidative damage caused by excess ROS may impair cell cycle progression and direct cells to death [16]. Thus, the ability of **1** and **2** to impair mitochondrial activity was also assessed via evaluation of ROS production. As shown in Figure 8, a 24-h exposure to **1** and **2** resulted in an increase in total intracellular ROS of about 4.6- and 5.2-fold vs. controls, comparable to that obtained with treatment with H_2_O_2_ as a positive control (about 4.8-fold).

## 3. Discussion

In the present paper, we have tested the cytotoxic effect on TNBC MDA-MB-231 cells of the ferrocene-containing HDACi **1** and its derivative **2**, already validated for their in vitro inhibitory activity on HDAC3 [9]. In the same article, biological assays were performed by exposing *Xenopus laevis* embryos and HDAC3-overexpressing cervical and colon cancer cell lines (HeLa, Ht-29, and HCT-113) to **1** and **2**. The results obtained highlighted the powerful activity of **1** vs. **2** in maintaining the acetylation of H4K12ac in the embryo and preventing HCT-113 cellular invasion at low concentration (1 μM). On the other hand, a moderate effect on the inhibition of cancer cell proliferation and colony formation was found for **1** at about ten-fold higher concentration whereas **2** was ineffective. Concerning compound **3**, the ferrocenium salt was found to affect HCT-113 colony formation poorly, with its activity being about 100-fold lower than that of **1** presumably due to its scarce intracellular uptake. Further confirmation of its meager inhibitory activity has been obtained here with MDA-MB-231 cells, thereby preventing additional molecular investigation on its biological effects.

Our data indicate that both compounds exert a cytotoxic effect on MDA-MB-231 cells prominently when administered at 70 μM concentration for 24 h. Interestingly, the dose–response curve appeared to be of the “U-shape” or hormetic type. Such curves have precedence in anticancer assays [17], being attributable to various causes such as the formation of intra- or extracellular complexes or the inactivation of intracellular pathways responsible of the biological activity at elevated concentrations of the drugs [18]. Further studies are required to clarify this issue as regards the compounds under study.

Treatment with **1** and **2** produced significant accumulation of MDA-MB-231 cells at the pre-G_0_/G_1_ cell cycle phase, which was accompanied by a decrease at the other phases, mostly S and G_2_/M. However, no phosphatidylserine externalization and caspase-8 activation was found using specific assays, thereby indicating that the cytotoxic effect of the two compounds might be ascribed to the onset of non-apoptotic cell death, as also reported for exposure of MDA-MB-231 cells to another ferrocene-containing class I HDACi (e.g., JAHA) [19] and exposure of other tumor cell model systems to the aryl-capped SAHA or depsipeptide [20]. It is worth mentioning that MDA-MB-231 cells possess a mutant p53, which sustains survival, and elevated levels of the apoptosis-suppressor phospholipase D, thereby being resistant to programmed cell death [21,22].

The most intriguing results on the mechanisms of action of **1** and **2** concern their ability to induce autophagy inhibition and ROS production/MMP dissipation. The data presented here suggest that **1** and **2** can be included in the list of those molecules that down-regulate autophagy, thereby depriving TNBC cells of such a pro-survival mechanism. In particular, flow cytometry results showed a massive reduction in AVO number and size when cells were treated with **1**, whereas exposure to **2** resulted in a slightly lower AVO accumulation and extension than that of control. When autophagy markers were evaluated electrophoretically, the amount of p62/SQSTM1 appeared to markedly increase after 24 h incubation with **1**, remaining somewhat higher than the control in the case of exposure to **2**. Moreover, there was a reduction in the accumulation levels of Beclin-1 following exposure to both HDACis. According to the data published by [23], an increase in the p62/Beclin-1 ratio (average values = 0.63 for control and 1.08 for **1**) is closely linked to the inhibition of the autophagic process. On the other hand, also in the case of cells exposed to **2**, the p62/Beclin-1 ratio showed a sizeable increase (average value = 1.18) although the intensity of fluorescence emitted by acridine orange-stained AVOs did not appear greatly modified with respect to control. It is known that acridine orange is taken up not only by autolysosomes but also by the amount of heterolysosomes present; however, it is not a good marker of the earlier autophagosomes [24]. Therefore, if the increase in p62/Beclin-1 ratio is indicative of autophagy inhibition, it is conceivable that the lack of prominent modifications in flow cytometry analyses might be due to the fact that the heterophagic activity in cells exposed to **2** remains unmodified or may even rise. Conversely, the data obtained after treatment with **1** suggest a generalized down-regulation of the intracellular degradation systems. Regarding the LC3 marker, the LC3-II/LC3-I ratio appeared to increase in MDA-MB-231 cells exposed to both HDACis vs. control. LC3-II accumulation is related to autophagosome maturation, which, once completed, results in the activation of autophagy and the degradation of LC3. As suggested by [24], up-regulation of the LC3-II/LC3-I ratio might be linked to a failure of autophagosome maturation and turnover likely due to a delay or impairment of the lysosomal degradation process, thus, further substantiating the inhibitory effect exerted by **1** and **2** on the occurrence of autophagy when administered to the TNBC cells under study.

Both treatments were effective in inducing the dissipation of MMP and an increase in the levels of ROS. It is known that ROS are by-products of mitochondrial respiration, which impairs intracellular signaling and also induces oxidative damage. Cells possess enzymatic and non-enzymatic antioxidant defense systems that counterbalance ROS accumulation and are involved in the maintenance of redox homeostasis [25]. The amount of intracellular ROS increases due to an imbalance of ROS production, removal, and extramitochondrial release. Conceivably, treatment of MDA-MB-231 cells with **1** and **2** induces oxidative stress likely due to the interruption of redox homeostasis from structural impairments of the mitochondria and/or loss of matrix solutes (e.g., glutathione) from an increase in their membrane permeability. Moreover, ROS production may further damage the mitochondria, thus initiating a vicious cycle leading to the exacerbation of ROS formation, mitochondrial dysfunction, and MMP collapse as reported in various papers [26,27], including the present one. It is also acknowledged that mitochondria play a crucial and complex role in autophagy. In particular, some authors [28,29] reported that the accumulation of MMP-deficient mitochondria impairs cellular autophagic activity and autophagosome turnover possibly through reduced lysosomal acidification, which ultimately leads to cell death. In light of the evidence produced in our work, it is conceivable that the cytotoxic activity of the HDACis tested on TNBC cells relies, at least partly, on the onset of oxidative damage and autophagy inhibition.

Further studies will be required to determine the precise cellular pathways through which **1** and **2** accomplish their TNBC cell death activity, particularly since our data suggest that the activity of compound **2** is conceivably HDAC independent. Nonetheless, our work on compounds **1** and **2** presents interesting aspects in that it expands the list of autophagy-regulating drugs and provides more insight into the role played by autophagy inhibition in breast cancer cell death. Interestingly, the HDACi **2** that was poorly active on different biological models (i.e., *Xenopus laevis* embryo cells and non-breast-derived malignant cancer cells, as reported by [9]) was equipotent to the parental molecule **1** toward TNBC cells, thus further underlining the importance of thorough biological characterization of bioinorganic HDAC probes in different cell model systems. A limitation of this study could be the high concentration (70 μM) of the HDACis, which was required to approximately halve the cell population in the in vitro system used. In fact, the IC_50_ value of most HDACis active on MDA-MB-231 cells is attested to be between 4 and 10 μM [20,30,31]. This differing potency might be explained by the specific HDAC selectivity of the compounds tested here. In fact, our results are in line with those obtained by Bian et al. [32] who demonstrated that solid tumor cell lines, such as MDA-MB-231, are less sensitive than hematologic cell lines to the anti-proliferative effect exerted by the selective HDAC2 and -3 inhibitor N-hydroxy-4-(1H-indol-3-yl) butanamide; its IC_50_ at 48 h for these cells being about 30 μM. Moreover, poor cell permeation and high lipophilicity might also lead to the high concentrations needed for the biological effect.

In summary, here we have shown that ferrocene-containing HDACis **1** and **2** are inhibitors of TNBC cell viability and growth in culture operating an impairment on cell autophagic process and mitochondrial function. The compounds studied may represent attractive and promising targets for following molecular modeling for anti-neoplastic agents in malignant breast cancer with the aim of their further optimization in terms of potentiation of cytotoxic activity and improvement of effectiveness.

## 4. Materials and Methods

### 4.1. Cell Culture and HDACis

MDA-MB-231 TNBC cells were cultured in d-MEM medium plus 10% fetal calf serum (FCS), 100 U/mL penicillin, 100 µg/mL streptomycin, and 2.5 mg/L amphotericin B (Sigma, St. Louis, MO, USA) at 37°C in a 5% CO_2_ atmosphere. The cells were detached from flasks with 0.05% trypsin-EDTA, counted, and plated at the necessary density for treatment once they had achieved 60%–80% confluency.

Compounds **1**, **2,** and **3** were synthesized as reported by [9], dissolved in dimethyl sulfoxide (DMSO) as stock solutions and stored at room temperature until use.

### 4.2. Cell Viability Assays

The in vitro cytotoxic activity was evaluated with (i) direct cell counting and (ii) the MTS-based CellTiter96^®^AQ_ueous_ One Solution Cell Proliferation Assay (Promega Italia, Milan, Italy) according to manufacturer’s instructions. Briefly, MDA-MB-231 cells in exponential growth were plated at a concentration of 5,500 cells/well in a 96-well plate and left to adhere overnight. Next, cells were treated with either 100 nM, 1, 10, 50, 70, or 100 µM of each compound or with DMSO vehicle for 24, 48, and 72 h and processed for the viability assay. Direct cell counting was performed in a Bürker chamber after trypan blue staining of trypsinized cells. For the MTS assay, after the addition of 20 µL of MTS reagent to each well, the absorbance of the dye was measured in an automated microplate reader at 490 nm. Cell viability ratios relate to the ratio between treated cells and untreated controls.

### 4.3. Flow Cytometry

Flow cytometric assays were performed according to [12]. The occurrence of apoptosis was checked with both the Annexin V-FITC kit (Canvax Biotech, Cordoba, Spain) which monitors the rate of externalization of phosphatidylserine, and the Vybrant^®^ FAM Caspase-8 Assay Kit (Molecular Probes, Eugene, OR, USA), which monitors the extent of activation of caspase-8, following the manufacturer’s instructions. Data were represented as dot plots using Flowing Software v.2.5.1., which discriminates normal cells (bottom left quadrant) from those undergoing early apoptosis (bottom right quadrant) or those undergoing late apoptosis/necrosis (top right quadrant).

The production of ROS was assessed with the ROS Detection Assay Kit (Canvax Biotech) following the manufacturer’s instructions. A H_2_O_2_-treated positive control was included in the analysis, and data were represented as histograms using Flowing Software v.2.5.1.

MMP was checked with the fluorescent dye JC1 (Molecular Probes), which is selectively internalized by mitochondria. In the case that of intact MMP and it polymerizes, the fluorescence emission shifts from green (~529 nm) to red (~590 nm). In the case of MMP dissipation and it remains as a monomer, the red/green fluorescence intensity ratio decreases. A positive control obtained by co-incubation of JC1 with 1 mg ionophore valinomycin (Sigma)/mL, which induces mitochondrial gradient dissipation, was included in the analysis. Data were represented as dot plots using Flowing Software v.2.5.1., which discriminates in the bottom quadrant the number of cells that undergo a loss of MMP.

Quantification of AVOs was enabled by staining with acridine orange, a cell-permeable fluorescent dye that is taken up, protonated, and sequestered in the acidic compartments. Cells were fixed with cold 70% ethanol and incubated with 100 µg acridine orange/mL (Sigma) for 20 min in the dark before analysis. Data were represented as dot plots using Flowing Software v.2.5.1., which discriminate cells with increased AVO accumulation in the top quadrant.

Cell cycle distributions were evaluated by propidium iodide staining; following pre-incubation (Triton X-100 and RNase A (Sigma)), they were analyzed using Weasel v.3.0.1. software.

All the preparations assayed contained both attached and floating cells, and all the analyses were performed in a FACSCanto apparatus (BD Biosciences, Franklin Lakes, NJ, USA).

### 4.4. Protein Blot

Electrophoretic analysis and immunoblots were performed following [20]. Lysates of control and treated cells were submitted to 13% SDS-PAGE and then protein transfer onto Hybond-ECL nitrocellulose membranes (GE Healthcare, Piscataway, NJ, USA) in a Novablot semidry apparatus (Pharmacia, Stockholm, Sweden). The primary antibodies used for immuno-detection were: rabbit polyclonal anti-Beclin-1 antibody (H-300, sc-11427, Santa Cruz Biotechnology, Dallas, TX, USA; working dilution 1:1000), rabbit polyclonal anti-LC3 antibody (L8918, Sigma; working dilution 1:750), rabbit polyclonal anti-p62/SQSTM1 (P0068, Sigma; working dilution 1:1000), and rabbit polyclonal anti-actin antibody (A5060, Sigma; working dilution 1:500). The latter antibody was used for a loading control. The secondary antibody was a horseradish peroxidase-conjugated anti-rabbit IgG antibody (NA934V, GE Healthcare; working dilution 1:2500). Protein bands were visualized in a Versadoc MP4000 molecular digital imaging system (Bio-Rad, Hercules, CA, USA) using the Immun-Star WesternC Chemiluminescent Kit (Bio-Rad), and analyzed by ImageJ software. Quantitation was performed after normalization of the signals with respect to those obtained with anti-actin antibody reaction.

### 4.5. Statistics

Statistics were checked through ANOVA test with SigmaStat 4.0 software (SYSTAT, San Jose, CA, USA). A *p*-value <0.05 was considered statistically significant.

## Abbreviations

HDACi	histone deacetylase inhibitor
SAHA	suberoylanilide hydroxamic acid
TNBC	triple-negative breast cancer
HER2	human epidermal growth factor receptor 2
GSK3β	glycogen synthase kinase 3 beta
MTS	3-(4,5-dimethylthiazol-2-yl)-5-(3-carboxymethoxyphenyl)-2-(4-sulfophenyl)-2H-tetrazolium
AVO	acidic vesicular organelle
SQSTM1	sequestosome-1
LC3	microtubule-associated proteins 1A/1B light chain 3B
MMP	mitochondrial transmembrane potential
ROS	reactive oxygen species
H4K12ac	acetylated histone H4 at lysine 12
JAHA	Jay Amin hydroxamic acid (*N*^1^-(ferrocenyl)-*N*^8^-hydroxyoctanediamide)
IC_50_	half maximal inhibitory concentration
D-MEM	Dulbecco’s Modified Eagle Medium
EDTA	ethylenediaminetetraacetic acid
DMSO	dimethyl sulfoxide
FITC	fluorescein isothiocyanate
SDS-PAGE	sodium dodecyl sulphate-polyacrylamide gel electrophoresis
ECL	enhanced chemiluminescence
ANOVA	analysis of variance

## References

[1] Seto E., Yoshida M. (2014). Erasers of histone acetylation: The histone deacetylase enzymes. Cold Spring Harb. Perspect. Biol..

[2] Garmpis N., Damaskos C., Garmpi A., Kalampokas E., Kalampokas T., Spartalis E., Daskalopoulou A., Valsami S., Kontos M., Nonni A. (2017). Histone deacetylases as new therapeutic targets in triple-negative breast cancer: Progress and promises. Cancer Genomics Proteomics.

[3] Eckschlager T., Plch J., Stiborova M., Hrabeta J. (2017). Histone deacetylase inhibitors as anticancer drugs. Int. J. Mol. Sci..

[4] Newbold A., Falkenberg K.J., Prince H.M., Johnstone R.W. (2016). How do tumor cells respond to HDAC inhibition?. FEBS J..

[5] Hudis C.A., Gianni L. (2011). Triple-negative breast cancer: An unmet medical need. Oncologist.

[6] Luparello C., Romanotto R., Tipa A., Sirchia R., Olmo N., López de Silanes I., Turnay J., Lizarbe M.A., Stewart A.F. (2001). Midregion parathyroid hormone-related protein inhibits growth and invasion in vitro and tumorigenesis in vivo of human breast cancer cells. J. Bone Miner. Res..

[7] Gartel A.L., Feliciano C., Tyner A.L. (2003). A new method for determining the status of p53 in tumor cell lines of different origin. Oncol. Res..

[8] Huovinen M., Loikkanen J., Myllynen P., Vähäkangas K.H. (2011). Characterization of human breast cancer cell lines for the studies on p53 in chemical carcinogenesis. Toxicol. In Vitro.

[9] Ocasio C.A., Sansook S., Jones R., Roberts J.M., Scott T.G., Tsoureas N., Coxhead P., Guille M., Tizzard G.J., Coles S.J. (2017). Pojamide: An HDAC3-selective ferrocene analogue with remarkably enhanced redox-triggered ferrocenium activity in cells. Organometallics.

[10] Hsieh H.Y., Chuang H.C., Shen F.H., Detroja K., Hsin L.W., Chen C.S. (2017). Targeting breast cancer stem cells by novel HDAC3-selective inhibitors. Eur. J. Med. Chem..

[11] Adhikari N., Amin S.A., Trivedi P., Jha T., Ghosh B. (2018). HDAC3 is a potential validated target for cancer: An overview on the benzamide-based selective HDAC3 inhibitors through comparative SAR/QSAR/QAAR approaches. Eur. J. Med. Chem..

[12] Librizzi M., Spencer J., Luparello C. (2016). Biological effect of a hybrid anticancer agent based on kinase and histone deacetylase inhibitors on triple-negative (MDA-MB-231) breast cancer cells. Int. J. Mol. Sci..

[13] Apel A., Herr I., Schwarz H., Rodemann H.P., Mayer A. (2008). Blocked autophagy sensitizes resistant carcinoma cells to radiation therapy. Cancer Res..

[14] Kanematsu S., Uehara N., Miki H., Yoshizawa K., Kawanaka A., Yuri T., Tsubura A. (2010). Autophagy inhibition enhances sulforaphane-induced apoptosis in human breast cancer cells. Anticancer Res..

[15] Mizushima N., Yoshimori T. (2007). How to interpret LC3 immunoblotting. Autophagy.

[16] He L., He T., Farrar S., Ji L., Liu T., Ma X. (2017). Antioxidants maintain cellular redox homeostasis by elimination of reactive oxygen species. Cell. Physiol. Biochem..

[17] Patel H., Chuckowree I., Coxhead P., Guille M., Wang M., Zuckermann A., Williams R.S.B., Librizzi M., Paranal R.M., Bradnerg J.E. (2014). Synthesis of hybrid anticancer agents based on kinase and histone deacetylase inhibitors. MedChemComm.

[18] Calabrese E.J., Baldwin L.A. (2003). Hormesis: The dose-response revolution. Annu. Rev. Pharmacol. Toxicol..

[19] Zhang J., Zhong Q. (2014). Histone deacetylase inhibitors and cell death. Cell. Mol. Life Sci..

[20] Librizzi M., Longo A., Chiarelli R., Amin J., Spencer J., Luparello C. (2012). Cytotoxic effects of Jay Amin hydroxamic acid (JAHA), a ferrocene-based class I histone deacetylase inhibitor, on triple-negative MDA-MB-231 breast cancer cells. Chem. Res. Toxicol..

[21] Zheng Y., Rodrik V., Toschi A., Shi M., Hui L., Shen Y., Foster D.A. (2006). Phospholipase D couples survival and migration signals in stress response of human cancer cells. J. Biol. Chem..

[22] Gurtner A., Starace G., Norelli G., Piaggio G., Sacchi A., Bossi G. (2010). Mutant p53-induced up-regulation of mitogen-activated protein kinase kinase 3 contributes to gain of function. J. Biol. Chem..

[23] Kara N.Z., Toker L., Agam G., Anderson G.W., Belmaker R.H., Einat H. (2013). Trehalose induced antidepressant-like effects and autophagy enhancement in mice. Psychopharmacology.

[24] Yin Z., Pascual C., Klionsky D.J. (2016). Autophagy: Machinery and regulation. Microb. Cell.

[25] Fan L.M., Li J.M. (2014). Evaluation of methods of detecting cell reactive oxygen species production for drug screening and cell cycle studies. J. Pharmacol. Toxicol. Methods.

[26] Payne B.A., Chinnery P.F. (2015). Mitochondrial dysfunction in aging: Much progress but many unresolved questions. Biochim. Biophys. Acta.

[27] Ježek J., Cooper K.F., Strich R. (2018). Reactive oxygen species and mitochondrial dynamics: The yin and yang of mitochondrial dysfunction and cancer progression. Antioxidants.

[28] Graef M., Nunnari J. (2011). A role for mitochondria in autophagy regulation. Autophagy.

[29] Rambold A.S., Lippincott-Schwartz J. (2011). Mechanisms of mitochondria and autophagy crosstalk. Cell Cycle.

[30] Zhang L., Zhang Y., Chou C.J., Inks E.S., Wang X., Li X., Hou J., Xu W. (2014). Histone deacetylase inhibitors with enhanced enzymatic inhibition effects and potent in vitro and in vivo antitumor activities. ChemMedChem.

[31] Chuang Y.F., Huang S.W., Hsu Y.F., Yu M.C., Ou G., Huang W.J., Hsu M.J. (2017). WMJ-8-B, a novel hydroxamate derivative, induces MDA-MB-231 breast cancer cell death via the SHP-1-STAT3-survivin cascade. Br. J. Pharmacol..

[32] Bian J., Luan Y., Wang C., Zhang L. (2016). Discovery of N-hydroxy-4-(1H-indol-3-yl) butanamide as a histone deacetylase inhibitor. Drug Discov. Ther..

